# Nitrogen and carbon stable isotope analysis sheds light on trophic competition between two syntopic land iguana species from Galápagos

**DOI:** 10.1038/s41598-022-21134-2

**Published:** 2022-10-07

**Authors:** Marco Gargano, Giuliano Colosimo, Paolo Gratton, Silvio Marta, Mauro Brilli, Francesca Giustini, Christian Sevilla, Gabriele Gentile

**Affiliations:** 1 PhD Program in Evolutionary Biology and Ecology, Department of Biology, University of Rome Tor Vergata, Rome, Italy; 2 Department of Biology, University of Rome Tor Vergata, Via della Ricerca Scientifica, snc, 00133 Rome, Italy; 3 Department of Environmental Science and Policy, Università Degli Studi Di Milano, via Celoria 10, 20133 Milano, Italy; 4 Institute of Environmental Geology and Geoengineering IGAG - CNR, Italian National Research Council, Area della Ricerca di Roma1, Via Salaria km 29.300, 00015 Monterotondo Stazione, Rome, Italy; 5 Galápagos National Park Directorate, Av. Charles Darwin - Puerto Ayora, Is. Santa Cruz, 200102 Galápagos, Ecuador

**Keywords:** Conservation biology, Stable isotope analysis

## Abstract

Coexistence between closely related species can lead to intense competition for resources. Stable isotope analysis (SIA) is a reliable tool to estimate the extent of species competition. We employed SIA to evaluate niche partitioning among two syntopic species of Galápagos land iguanas: *Conolophus subcristatus* and *C. marthae*. Samples were collected on Wolf Volcano, Isabela Island, where *C. marthae* is endemic and syntopic with *C. subcristatus*. We determined δ^13^C and δ^15^N ratios and described the isotopic niche of each species using corrected standard ellipse area (SEA_c_). We tested for differentiation between the isotopic niches, while controlling for sex, body size, spatial location of samples and mean annual primary productivity at capture points, using bivariate linear models. Despite the extensive overlap of the isotopic niches, we found species and sex to be a significant, interacting predictor of a sample’s location in the δ^13^C, δ^15^N space, indicating the existence of niche partitioning mechanisms acting between species and sexes. We also found that body size and productivity at the capture points, compounded with yet undetermined spatial effects, explain ca. 75% of the differences observed between species and sexes, providing evidence for differential microhabitat and food-items usage. Our study provides essential baselines for evaluating conservation actions for *C. marthae,* such as the potential translocation to a sanctuary area free of competition from *C. subcristatus*.

## Introduction

Competition is a critical determinant of population dynamics and community structure^1–3^. Closely related species that diverged in allopatry may retain their common ancestral niche and be expected to competitively exclude each other when they come into secondary contact^4^. Under these circumstances and given appropriate conditions^5^, natural selection acting on one or both species may favor traits that reduce niche-overlap and, therefore, competition^6^. This mechanism has been often called into question to explain patterns of coexistence between syntopic (*sensu* Rivas^7^) closely related species competing for limited resources^8–12^. On the other hand, neutral models of community evolution (“community drift”)^13–15^ may also explain niche divergence and, ultimately, coexistence between sister species. These neutral mechanisms may be expected to be particularly important where community structures are strongly limited by incomplete dispersal, as is typically the case for communities of terrestrial vertebrates living in oceanic islands^16^. Such communities are shaped by complex patterns of extinction and recolonization^17^, and species that happen to coexist on one island may have well spent large portions of their evolutionary past confined into different islands, experiencing different biotic and abiotic conditions and, hence, selective pressures^17^.

Beyond its fundamental importance as a key concept of animal ecology, a better understanding of competitive dynamics between co-occurring, closely related species can contribute important insights to the conservation of interacting endangered species. One such case involves two species of Galápagos land iguanas: *Conolophus subcristatus* and *C. marthae*. While the former is found on seven islands across the Galápagos Archipelago, namely Santa Cruz, Plaza Sur, Isabela, Fernandina, Seymour Norte (introduced), Baltra (repatriated) and Santiago (reintroduced;^18^), the latter is only found in a very small high-altitude area (*ca.* 25 km^2^) on Wolf Volcano, Isabela Island^19^. At this site, the only known population of *C. marthae* is syntopic with a population of *C. subcristatus*^20^. *Conolophus marthae* has been recently described to science^21^ and, shortly after, assessed as Critically Endangered in the IUCN Red List of Threatened Species^19^. The main justifications for this assessment include small population size, scarce recruitment, introduced predators, limited distribution, and the potential for intense competition with *C. subcristatus*^19–21^.

While new and important information on the ecology and biology of *C. marthae* are constantly accreting, very little is known about its dietary habits and trophic preferences, but some preliminary evidence, obtained indirectly from analyzing the chemical composition of femoral pore secretions, suggest they could be partially different from those of *C. subcristatus*^22^, for which a generalist trophic behavior has been described^23,24^. Moreover, field observations indicate differential microhabitat use, hinting towards the use of different trophic resources^25^. Molecular phylogeny indicates that the divergence of *C. marthae* and *C. subcristatus* started ca. 1.5 million years ago^26^, when the island of Isabela had not yet formed, and it is still unknown when these two species colonized Isabela and how long they existed in sympatry. As intense competition for trophic resources may lead to the extinction of the least competitive species^27,28^, a thorough exploration of the trophic ecology of *C. marthae* and *C. subcristatus* populations in syntopy is essential for guiding conservation actions^29,30^.

Stable isotope analysis (SIA) is an effective and widely applicable tool to characterize the trophic niche of syntopic species^31–33^. The isotopic composition of animal tissues is known to reflect the isotopic composition of their trophic resources^34–36^, with differences in diet composition translating into differences in the stable isotope composition^31,37^. In this work, we used isotope ratios to evaluate the dietary overlap between the syntopic populations of *C. marthae* and *C. subcristatus*. We determined δ^13^C and δ^15^N isotopic compositions in nail-keratin sampled from individuals of these two species on Wolf Volcano. We first estimated the degree of overlap between the isotopic spaces occupied by individuals of the two species by computing standard ellipse areas (SEAc) and assessed trophic niche segregation while controlling for individuals’ sex. On the one hand, measuring the extent of isotopic niche overlap contributes to the evaluation of the potential for competitive interaction between *C. marthae* and *C. subcristatus*. On the other hand, rejecting the null hypothesis of no differentiation in isotopic composition between species, would strongly argue for some mechanisms of niche partitioning to act between the two species. We then used a variance partitioning approach to assess how much ecologically relevant predictors such as body size and mean primary productivity at the capture points, compounded with unmeasurable variables linked to spatial location itself, explained variation in isotopic profiles and the differences between species and sexes.

## Materials and methods

### Study area and sampling

We captured 60 individuals of *C. marthae* (38 males, 22 females) and 58 individuals of *C. subcristatus* (33 males, 25 females) from June 14 to June 23 2014. We sampled the northwestern slope of Wolf Volcano at an altitude of 1488–1659 m asl, within an area no larger than 2 km^2^. Individuals were hand-grabbed or captured using noosed telescopic poles. June is a transitional month, between the warm/wet season (typically occurring between January and May) and the cold/dry season (approximately from June to December^38,39^).

For each captured individual, we collected nail-keratin samples and recorded GPS coordinates of the capture point, snout-to-vent length (SVL; in cm), mass (in kg) and sex. According to the habitat classification in^40^, 114 collected individuals (96.6% of the total) were captured in patches of “deciduous shrubland”, while only 2 *C. marthae* and 2 *C. subcristatus* were captured in a different habitat (evergreen seasonal forest and shrubland). Other habitat types in this area include deciduous forest, tallgrass vegetated areas, and evergreen forest on the western side of the volcano^40^. All captured individuals were classified as adults based on morphological characteristics. In compliance with the Galápagos National Park regulation, we minimized the nail-keratin amount sampled, taking three to four toenails from each individual, using sterilized bolt cutters. Nail-keratin samples were immediately placed into 1.5 ml polypropylene collection tubes. Sixty individuals were identified as having been captured and tagged in previous field expeditions by our research group^19–21^. All other captured iguanas were assigned a new ID and were tagged with a passive integrated transponder (PIT). To counter the illegal trade of Galápaganian wildlife^41^, in agreement with the Galápagos National Park, we decided not to disclose the exact GPS coordinates of the capture points here and avoid publishing a detailed map of our sampling.

### Stable isotopes analysis (SIA)

In animal ecology, SIA is performed by analyzing the ratios between the two main stable isotopes of carbon (^12^C and ^13^C) and nitrogen (^14^N and ^15^N) respectively, which can then be compared among individuals, species, or populations^34–36^. As animal tissues are generally enriched in ^13^C by only ≈ 1‰ relative to their food^34,36^, the distribution of δ^13^C in a sample of individuals is typically used to estimate between-individuals variation in the diet of the population they belong to. In contrast, the tissues of animals are enriched in ^15^N by ≈ 3–4‰ compared to their food source, and δ^15^N is widely used to estimate the relative trophic level of individuals and species^35,36^. Intuitively, the differences between the isotopic composition of food sources and that of animal tissues, indicated through the Trophic Discrimination Factor (TDF), can vary between species, and the values provided above should be considered indicative^42^. The turnover rates of stable isotopes vary largely among tissues, with metabolically more active tissues reflecting diet within a few weeks, and less active tissues being informative of dietary habits of the last several months^43,44^. Differences in δ^15^N and δ^13^C from different tissues from a single iguana individual have been described and interpreted as informative on diet composition in different time periods^45^. Inert tissues, such as the keratin in hair or nails, provide long-term information on consumed food sources^46^. For example, it has been demonstrated that, for turtles’ claws, a change in diet requires about 6 and 12 months to be reflected by a change in δ^15^N and δ^13^C, respectively^47^, while it takes 3 to 4 months in endothermic Sauropsida (birds^48^).

To determine the isotopic composition of carbon and nitrogen, toenail samples were cleaned using distilled water to remove any sources of contamination. Nail-keratin fragments were freeze-dried (− 55 °C) and then ground into a fine, homogeneous powder with an agate pestle and mortar. They were then placed into small tin capsules (4 × 6 mm). For each individual, a pair of samples of 0.2–0.3 mg was analyzed, using continuous-flow isotope ratio mass spectrometry (Thermo Flash 1112 Elemental Analyzer coupled to a Finnigan Delta Plus mass spectrometer) at IGAG (Istituto di Geologia Ambientale e Geoingegneria, CNR). Isotopic compositions were expressed in the usual δ notation, which represents the relative deviation in part per thousand of the heavy isotope/light isotope ratios of the sample from the same ratio of a reference standard. Isotope data were normalized to the V-PDB (Vienna Pee-Dee Belemnite) scale for carbon and AIR (atmospheric air) scale for nitrogen using IAEA standards (CH-6, CH-7 USGS-24 for carbon, and N-1, N-2, USG25 for nitrogen). Based on repeated measurements of laboratory standards, the analytical error was < 0.3 ‰ for both carbon and nitrogen.

### Data analysis

To characterize the isotopic niche of each species and sex, we calculated standard ellipse areas corrected for small sample size (SEA_c_), as described by Jackson and colleagues^49^. SEA_c_ was developed to represent the core of the isotopic niche of a sampled population, and we used these metrics to measure niche overlap between species and sexes as the proportion of overlap between ellipses. The entire procedure was carried out using the “SIBER” R-package^49^.

We tested for differences in carbon and nitrogen stable isotope profiles between species and sexes by fitting a bivariate linear model, with δ^13^C and δ^15^N as joint response variables and species, sex and their interaction as predictors. To evaluate the effect of individual morphology, habitat features and spatial distribution of the samples, we fitted Generalized Additive Models (GAMs) including parametric terms for body-size and mean primary productivity at the capture point, and a spatial tensor product on the *X* and *Y* coordinates (UTM/WGS-84 Zone 15S) of the capture points accounting for unmeasured variables related to the spatial distribution of samples. We expressed body size as snout-to-vent-length (SVL). We chose SVL rather than mass to express body size because the former represents a more stable measure, not strongly affected by reproductive status, fat storage and digestive state of the animal^50–54^. We measured primary productivity as Normalized Difference Vegetation Index (NDVI), using Copernicus Sentinel-2 10 m resolution images, freely available on the cloud service Google Earth Engine (https://earthengine.google.com/). We calculated the mean NDVI for a 50-m radial buffer around the capture point of each individual averaged on a 3-year period (2019–2021), after masking for clouds (*i.e.,* excluding from the averaging all pixels where clouds were detected in a given image). Before fitting our models, both SVL and NDVI were standardized to zero mean and unit standard deviation using the *scale()* function from package “base”^55^ in order to obtain more easily interpretable coefficients. We preliminarily investigated whether these two variables differed between species and sexes by fitting two linear models with scaled SVL and NDVI as response variables and species and sex as predictors. To assess spatial aggregation of the samples, we conducted a multiple regression on distance matrices using the *MRM()* function in the “ecodist” R package^56^ with the Euclidean distance matrix computed from *X* and *Y* coordinates of the capture points as response variable and the dissimilarity matrices of species and sex as predictors (where 0 indicates matching and 1 indicates not-matching).

To investigate the contribution of ecologically-relevant variables (size, productivity and space) in defining the isotopic differences observed between species and sexes, we used a variance partitioning approach based on partial correlation analyses^57^, which we implemented by adapting the *ecospat.varpart()* function from package “ecospat”^58^. To this end, we fitted two additional models: first, a “full” bivariate generalized additive model (GAM) with δ^13^C and δ^15^N as joint response variables and species, sex, body size and mean productivity at the capture points as parametric predictors and a tensor product smooth interaction of geographic coordinates to adjust for the spatial effect of samples distribution; second, an analogous model excluding species and sex from predictors. We then compared these models with the previously described model considering species and sex only and computed the portions of deviance (i) uniquely explained by species + sex, (ii) uniquely explained by size + productivity + spatial tensor product, (iii) jointly explained by the two sets of predictors, and (iv) left unexplained^59^.

We fitted all models using the R function *gam()* from package “mgcv”^60^. For each model, we also checked that distribution of residuals was consistent with assumptions of normality and homoscedasticity. We used Likelihood Ratio Test (LRT) as implemented in the R function *anova()* from package “stats”^55^ to test for the significance of individual predictors in GAM models and *F* as test statistics to compute *P*-values for linear models with SVL and NDVI as response variables. Non-significant interactions were removed from our final models. We checked for model stability by re-fitting each model 200 times on random sub-samples with 90% of the data and visually assessing the robustness of the coefficient estimates. We did not find evidence of instability for any of our models (for details see SM Fig. 1; SM Fig. 2; SM Fig. 3).

### Study design

This study was designed, carried out and reported following the ARRIVE guidelines (https://arriveguidelines.org) according to Percie du Sert^78^.

### Ethics approval

The handling and sampling of the animals were carried out according to a protocol that minimized the stress of the animals. These techniques were approved by the University of Rome “Tor Vergata” ethics and animal handling protocol and following the guidelines of the European Community and with the approval of the Directorate of the Galápagos National Park (GNPD). The GNPD does not have a specific ethics committee. However, the GNPD is responsible for the administration of the research (including the issuing of research permits) carried out in the protected areas of the Galápagos, as administrator of these areas and representative of the National Environmental Authority. The GNPD has granted a research permit to G.G. for this project. The samples were exported and imported with GNPD and CITES permits issued to G.G.

## Results

**Figure 1:**
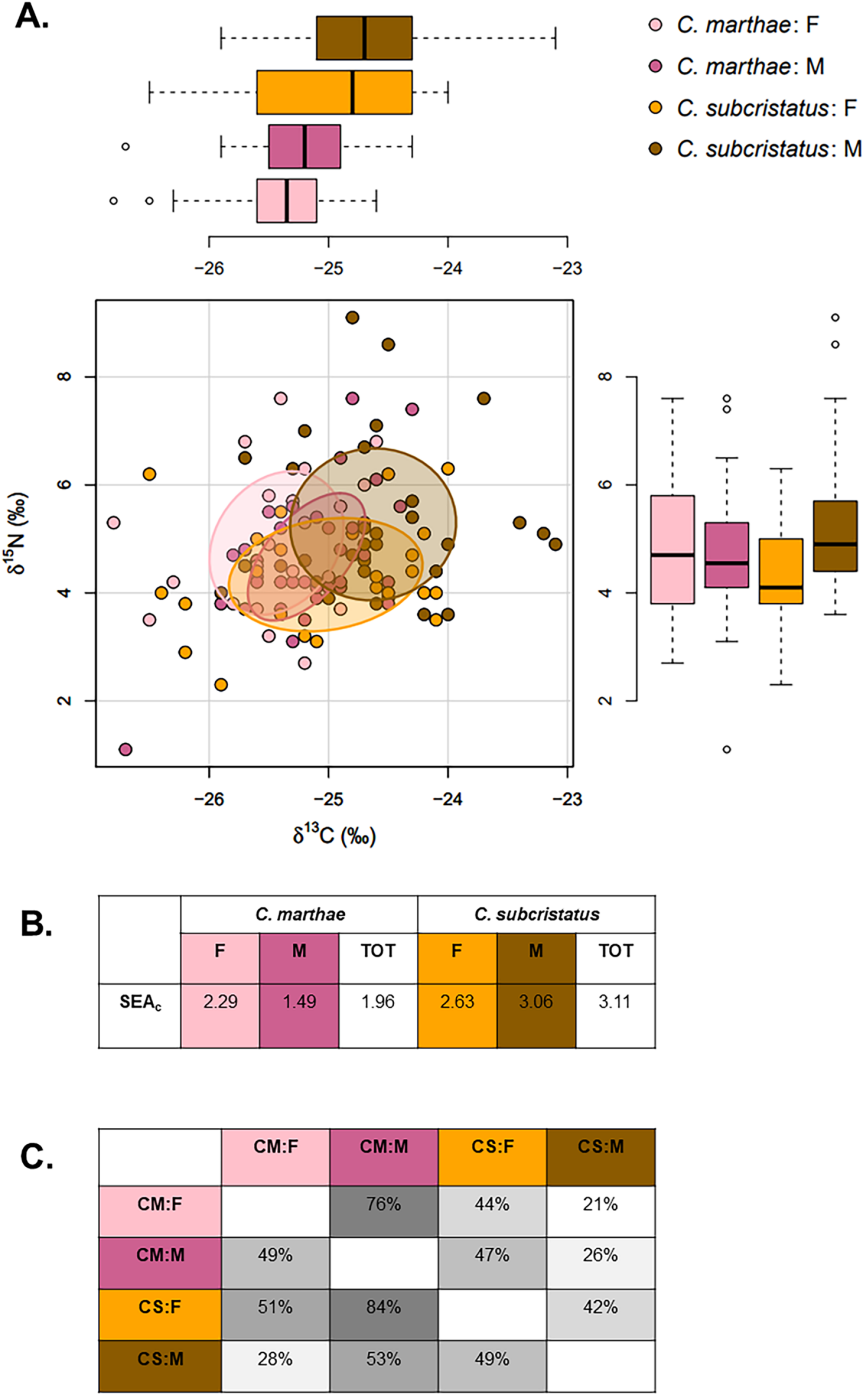
Carbon and nitrogen isotope niche of *C. subcristatus* and *C. marthae* males and females. In (**A**) each dots represents an individual. Solid lines enclose standard ellipse areas with sample size correction (SEA_c_). Marginal boxplots show the distribution of δ ^13^C and δ ^15^ N for each combination of sex and species. Color codes are shown in the top right corner. (**B**) Shows estimated SEA_c_ for each species and sex. (**C**) Shows pairwise overlap (expressed as percentage) between each combination of sex and species (CM:F = *C. marthae* females, CM:M = *C. marthae* males, CS:F = *C. subcristatus* females, CS:M = *C. subcristatus* males). Note that overlap is not symmetrical because values indicate the percentage of overlapping area over each ellipse. Intensity of grey is proportional to overlap.

We found that SEA_c_ was larger for *C. subcristatus* than for *C. marthae* (Fig. 1A–B). The overlapping portions of SEA_c_ were 65% and 41% for *C. marthae* within *C. subcristatus* and *C. subcristatus* within *C. marthae*, respectively. Both sexes of *C. subcristatus* had larger SEA_c_ than the corresponding sex of *C. marthae* (Fig. 1A–B)*.* At intraspecific level, we found higher levels of overlap between sexes for *C. marthae* (76% for males and 49% for females; Fig. 1C) than for *C. subcristatus* (42% for males and 49% for females; Fig. 1C).

Results of models fitted using δ^13^C and δ^15^N as response variables are reported in Table 1. Model 1 (Table 1), which only included species, sex and their interaction as predictors, indicated that species and sex independently influenced δ^13^C, with values in *C. marthae* being lower than those in *C. subcristatus* and higher in males than females (Fig. 1A), while δ^15^N depended on the interaction between sex and species, with similar values for males and females in *C. marthae* and clearly higher values for males than females in *C. subcristatus* (Fig. 1A). In Model 2 (Table 1), where size, productivity and space were added, only species showed a statistically significant effect on δ^13^C, while species and sex had no effect on δ^15^N, and body size and primary productivity at capture points strongly influenced the relative position of each individual in the δ^13^C, δ^15^N bi-plot space. The spatial tensor did not provide a statistically significant contribution to the model for δ^13^C, but it did for δ^15^N. The same pattern was found with Model 3 (Table 1).

**Table 1:** Estimated models coefficients for the two response variables, δ^13^C and δ^15^N.

	Estimate	SE	χ^2^	*Z*	*df*	*P*
**Model 1: (**δ^**13**^**C,** δ^**15**^**N) ~ Species + Sex**						
δ^**13**^**C**						
Intercept	− 25.483	0.109	–	–	–	–
Species (*C. subcristatus*)	0.499	0.114	19.130	4.374	1	<< 0.001
Sex (male)	0.325	0.117	7.780	2.789	1	0.005
δ^**15**^**N**						
Intercept	4.903	0.255	–	–	–	–
Species (*C. subcristatus*)	− 0.549	0.348	–	− 1.580	–	–
Sex (male)	− 0.223	0.319	–	− 0.700	–	–
Species (*C. subcristatus*): Sex (male)	1.126	0.444	6.423	2.530	1	0.011
**Model 2: (**δ^**13**^**C,** δ^**15**^**N) ~ Species + Sex + SVL + NDVI + te (*X*, *Y*)**						
δ^**13**^**C**						
Intercept	− 25.266	0.103	–	–	–	–
Species (*C. subcristatus*)	0.243	0.119	4.167	2.041	1	0.041
Sex (male)	0.174	0.105	2.733	1.653	1	0.098
SVL	0.307	0.055	31.089	5.576	1	<< 0.001
NDVI	− 0.120	0.056	4.578	− 2.140	1	0.032
te (X, Y)	–	–	4.171	–	3	0.339
δ^**15**^**N**						
Intercept	5.011	0.239	–	–	–	–
Species (*C. subcristatus*)	− 0.612	0.348	–	− 1.760	–	–
Sex (male)	− 0.166	0.284	–	− 0.590	–	–
Species (*C. subcristatus*): Sex (male)	0.729	0.408	3.188	1.790	1	0.074
SVL	0.356	0.103	11.906	3.450	1	0.001
NDVI	− 0.234	0.107	4.833	− 2.200	1	0.028
te (X, Y)	–	–	25.833	–	3	<< 0.001
**Model 3: (**δ^**13**^**C,** δ^**15**^**N) ~ SVL + NDVI + te (*X*, *Y*)**						
δ^**13**^**C**						
Intercept	− 25.041	0.051	–	–	–	–
SVL	0.365	0.051	50.472	7.100	1	<< 0.001
NDVI	− 0.135	0.057	5.683	− 2.380	1	0.017
te (X, Y)	–	–	5.725		3	0.126
δ^**15**^**N**						
Intercept	4.814	0.094	–	–	–	–
SVL	0.340	0.095	12.756	3.570	1	< 0.001
NDVI	− 0.258	0.105	6.033	− 2.460	1	0.014
te (X, Y)	–	–	30.947	–	3	<< 0.001

The table shows the output of the three fitted models: Model 1 is the bivariate linear regression with species and sex as predictors; Model 2 is the bivariate generalized additive model (GAM) with species, sex, body size (scaled SVL), productivity at the capture points (scaled NDVI) and a spatial tensor product smooth (te (*X*, *Y*)) as predictors; Model 3 is the bivariate generalized additive model (GAM) including only body size, productivity of the capture point and the spatial tensor product smooth as predictors. The table reports the estimated model value (Estimate), standard error (SE), degrees of freedom (*df*), the χ^2^ statistic for LRT with the corresponding *P*-values (*P)* and the *z*-score (*z*). The interaction between Species and Sex was not a significant predictor of δ^13^C (χ^2^ = 0.386, *P* = 0.535) and was therefore removed from the final models. *P*-values for intercept and for main effects included in an interaction do not have a meaningful interpretation and are not shown.

**Figure 2:**
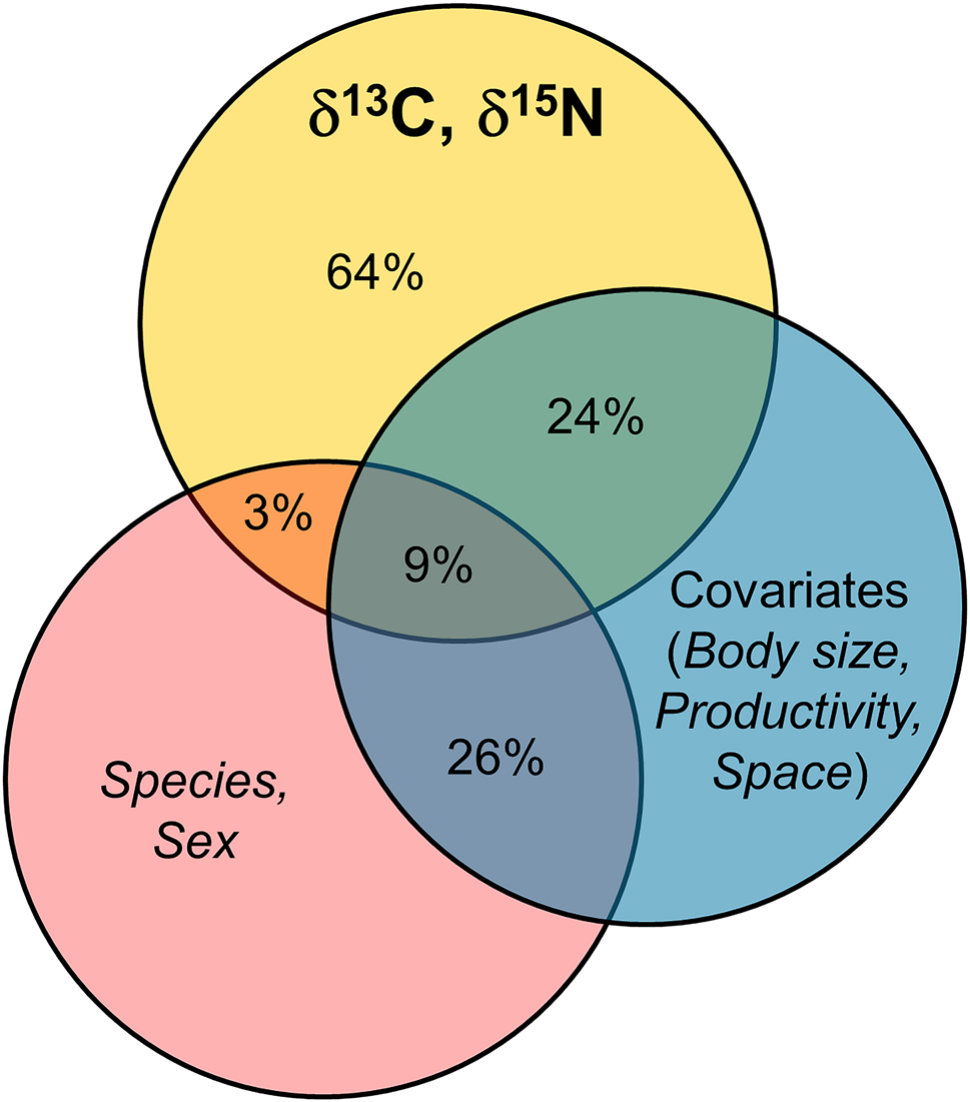
Venn diagram illustrating explained deviance estimated by variance partitioning. The yellow circle on top represents the total deviance of the response variables, whereas the red (bottom left) and blue (right) circles represent sets of predictors (species + sex and body size + productivity + space, respectively). The total deviance of each circle is set to unity. The area of the intersection between each of the predictors’ circles (red and blue) and the response (yellow) circle represents the deviance in the response variables that is explained by each set of predictors. The intersection of all circles is the variance jointly explained by the two sets of predictors. The intersection between the red and the blue circles represents the correlation between the two sets of predictors. The area of the yellow circle not overlapped by any other circle shows the proportion of response deviance not explained by the predictors.

Results of variance partitioning analysis are illustrated in Fig. 2. Our predictors collectively explained 36% of the deviance in the response (i.e., 64% of the total deviance was left unexplained by our analysis). Species and sex together explained only 12% of the response deviance, while size, productivity, and the spatial tensor, as a whole, explained 33%. Importantly, 9% of the deviance was jointly explained by the two sets of predictors. The latter result shows that the three quarters (9% on 12%) of the variation in isotopic composition observed between species and sexes may be explained by differences in size, productivity of the habitat they exploit, and some yet unmeasured spatial variable.

Consistently, results of univariate linear models with scaled SVL and NDVI as response variables (Table 2) showed that body size strongly differed between species and sexes (*P* < 0.001;* P* = 0.006 respectively), with *C. marthae* being, on average, smaller than *C. subcristatus* and males being, on average, larger than females (SM Fig. 4). The productivity of the capture points was higher for *C. marthae* than for *C. subcristatus* (*P* = 0.018; Table 2; SM Fig. 4) while it did not differ between sexes (Table 2; SM Fig. 4). Multiple regression on distance matrices indicated that species were slightly aggregated (*P* = 0.002) whereas we did not find evidence of spatial aggregation of individuals of the same sex (*P* = 0.939).

**Table 2:** Estimated coefficients for the two univariate linear models with SVL and NDVI, respectively, as response.

	Estimate	SE	*F*	*df*	*P*
**SVL**					
Intercept	− 0.613	0.163	–	–	–
Species (*C. subcristatus*)	0.643	0.171	14.098	1	< 0.001
Sex (male)	0.493	0.175	7.936	1	0.006
**NDVI**					
Intercept	0.117	0.172	–	–	–
Species (*C. subcristatus*)	− 0.435	0.181	5.796	1	0.018
Sex (male)	0.161	0.185	0.759	1	0.386

The table shows the estimated model value (Estimate), standard error (SE), degrees of freedom (*df*), the *F* statistic and corresponding *P*-values (*p*).

## Discussion

In this study, we used stable isotopes analysis to evaluate the potential for trophic competition between the only known population of the Galápagos Pink Land Iguana, *C. marthae*, and the syntopic population of the closely related and more widely distributed *C. subcristatus* on Wolf Volcano, Isabela Island. Our results rejected the null hypothesis of no difference in carbon and nitrogen stable isotopes profiles between species (Table 1—Model 1), indicating some degree of trophic niche partitioning. We also found that *C. marthae* occupies a smaller isotopic niche than *C. subcristatus*, with 65% of the standard ellipse area falling within the SEA_c_ of *C. subcristatus*. The similar δ^15^N recorded for the two species (Fig. 1A, Table 1—Model 1) indicate that they occupy similar trophic levels, consistently with previous reports indicating that both species primarily feed on vegetation, as typical of most large iguanas^23,24,61,62^. Importantly, males and females show larger isotopic space overlap in *C. marthae* than in *C. subcristatus* (Fig. 1A–C, Table 1—Model 1), suggesting that differentiation between the isotopic niches of males and females contributes to the larger overall niche occupied by *C. subcristatus*. Comparing the standard ellipses obtained from raw data (Fig. 1A) and those from the residuals of a model accounting for size, productivity and space (SM Fig. 5), it appears that, while the difference between sexes in residual δ^13^C of *C. subcristatus* is much smaller than in the raw data, the difference in δ^15^N is similar, implying that potential variation in trophic resource consumption between male and female *C. subcristatus* is not explained by differences in size or occupied habitat and may directly reflect sex-specific resource selection. A detailed characterization of δ^13^C and δ^15^N content of different plant species from the top of the volcano would be necessary to better investigate this pattern.

Previous data hinting at trophic resource partitioning between *C. marthae* and *C. subcristatus* were provided by a recent study indicating that differences in chemical composition of femoral pore secretions extracted from these two species could be associated with a difference in species diet^22^. Trophic niche-partitioning might be achieved by two non-mutually exclusive mechanisms. First, individuals of the two species could feed on different trophic resources in the overlapping distribution area. Second, they could feed in different habitats or microhabitats and consequently use different types of food^1–3^.

Both strategies are supported by our data. On the one hand, we found that (i) the capture locations of the two species were spatially aggregated, (ii) the two species were captured at locations differing in primary productivity (Table 2; SM Fig. 4) (iii) productivity at capture points is a significant predictor of both δ^13^C and δ^15^N (Table 1—Model 2 and Model 3) and (iv) unmeasured features of the space (measured by the spatial tensor product) have a significant effect on δ^15^N (Table 1), indicating selection of different microhabitats as a likely niche partitioning mechanism. Recent field observations also support a differential microhabitat use for these two species, with *C. marthae* that seems to preferentially occupy more shaded areas (Gentile, unpublished data), and different use of habitats or microhabitats by the two species was proposed as a potential mechanism contributing to prevent interspecific hybridization between *C. marthae* and *C. subcristatus* on Wolf Volcano^25^. On the other hand, resource selection may also contribute to differences in isotope niches. For example, while C_3_ plants have a δ^13^C ranging from − 22‰ to − 30‰, C_4_ show values ranging from − 10‰ to − 14‰^63^, and CAM (Crassulacean Acid Metabolism) from − 10 to − 20‰^64^. Lower δ^13^C values in *C. subcristatus* (Fig. 1A) could therefore indicate that the diet of this species includes a marginally larger portion of C_4_ and/or CAM plants than the diet of *C. marthae*. Consistent with this view, *Opuntia* cactus, which were previously reported to constitute a large portion of the diet of *C. subcristatus* and *C. pallidus*^65,66^ during the dry season, is a constitutive CAM species^67^, relatively rare inside the area of distribution of *C. marthae*, but abundant on the eastern side of Wolf Volcano. While this area is outside the core area of *C. marthae*, it is inhabited by *C. subcristatus*. It is not uncommon that when two species compete for common resources, the dominant species (often the larger one) could displace the less competitive one to a suboptimal trophic niche^68^. Therefore, the current situation on Wolf Volcano could be the result of a competitive process that has led *C. marthae* to use a suboptimal array of trophic resources. We foresee that estimates of the time at which these two species colonized Isabela and then reached Wolf Volcano to establish their current syntopy (which can be obtained from population genomics analyses) will help to clarify the nature of the interaction we are currently observing.

Admittedly, the absence of information about the trophic discrimination factor (TDF) of species may introduce inaccuracy in the interpretation of isotope data, in particular for δ^13^C^42^. The TDF-δ^13^C and to a lesser extent TDF-δ^15^N can be affected by metabolic routing, heterogeneity in isotope ratios and differences in macronutrient content among food sources^69^. Mean TDF-δ^13^C and TDF-δ^15^N estimates provided for the skin of three species of *Cyclura* ranged from + 2.8 to + 5.5‰ and + 5.8 to + 6.2‰, respectively^45^. If TDF-δ^13^C and TDF-δ^15^N of *Conolophus* species are similar to those described in *Cyclura*, we note that differences in δ^13^C and δ^15^N between the two *Conolophus* species are well within the range of variation of TDFs. Conceivably, despite differences in macronutrients and metabolic routing ultimately lead back to dietary characteristics^42^, we cannot rule out a possible role of unknown genetic or environmental factors.

Our data hint at resource partitioning operating at an intraspecific level too, with males of both species showing higher δ^13^C than females (Fig. 1A) and *C. subcristatus* males showing higher δ^15^N than their conspecific females (Fig. 1A). Intraspecific niche partitioning mechanisms are widespread in animal populations and can be interpreted as a strategy to reduce intersexual competition for food resources^70^. The isotopic pattern observed in our data is likely to reflect behavioral differences between sexes. It is well known that, during the reproductive season, approximately between January and May, on Fernandina Island, *C. subcristatus* males exhibit a territorial behavior, defending areas that constitute the core part of the mating area, while the females of the species shift between territories defended by different males^71^. Males reduce feeding during the mating season and food resources inside or near their territories are primarily necessary for females^71^. After mating, females leave these areas to reach suitable nesting sites, while males remain in their territories if food is still abundant or they disperse, looking for alternative feeding areas^71^. These observations, therefore, indicate that both temporal and spatial mechanisms act to reduce intersexual competition for trophic resources in *C. subcristatus* population on Fernandina Island. As δ^15^N of consumers generally increase with starvation^72^, if the same behavioral mechanisms acted in *C. marthae* and *C. subcristatus* populations on Wolf Volcano, males would show higher δ^15^N during the period for which our data are informative (which can be very approximately estimated to encompass the months from January to June). Interestingly, our results suggest that this explanation might be reasonable for *C. subcristatus* population on Wolf Volcano as males of the species showed δ^15^N values higher than those of conspecific females (Fig. 1A), while this difference was not revealed for *C. marthae* (males and females were characterized by similar δ^15^N values; Fig. 1A), suggesting that this behavior may not be shared by the two syntopic populations.

**Figure 3:**
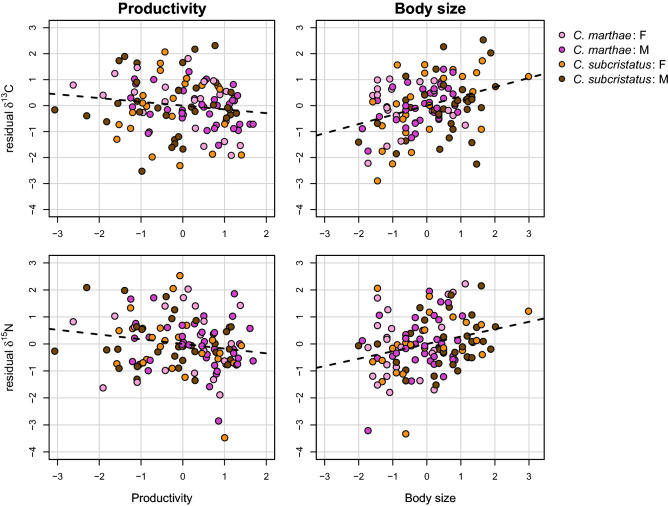
Visualisation of the marginal effects of productivity (left) and body size (right) on δ ^13^C (top) and δ ^15^N (bottom). Residuals of reduced models (identical to Model 2 in Table 1, but with the variable of interest excluded) are plotted against the variable of interest. Each dot represents a single individual. Color codes are shown in the top right corner.

A large portion of the variance observed in δ^13^C and δ^15^N between species and sexes can be explained in terms of body size, productivity, and relative location of the capture points (Fig. 2). Moreover, these ecological variables provided the highest independent contribution to explaining δ^13^C and δ^15^N recorded in the samples (Fig. 2). We found that, other predictors being equal, larger individuals show higher levels of both δ^13^C and δ^15^N (Table 1, Fig. 3), suggesting that they potentially feed on a different sweet of resources. It is not uncommon to find a positive relation between body size and trophic level in natural populations^73,74^. Larger individuals often have a competitive advantage over smaller ones, allowing them to integrate their diet with trophic resources of animal origin that could not be available for smaller individuals. Accordingly, Hanson and colleagues^75^ found a positive relationship between ^13^C and SVL in *Crocodylus porosus*, suggesting that individuals of different body size are linked to different primary resources. Our data, therefore, also suggest that the most intense competition may happen between individuals of the same age or at least class size, but we lack the appropriate resolution in our data to analyze this aspect in more detail.

We also found a negative relation between the NDVI of samples location and both δ^13^C and δ^15^N (Fig. 3). On the one hand, different availability of C3, C4 and CAM plant resources in areas with different productivity may contribute to the differences in δ^13^C. On the other hand, as animal tissues are enriched in both δ^13^C and δ^15^N relative to their diet, a direct explanation of this pattern may involve a higher consumption of animal food by those iguanas that occupy less productive microhabitats compared to those that were captured in more productive habitats. Interestingly, *Conolophus marthae* individuals were mostly found in areas with higher levels of NDVI, if compared with *C. subcristatus* (SM Fig. 4). More vegetated areas also offer better shelter opportunities. Evidence in the field suggest that *C. marthae* may prefer shaded areas whereas *C. subcristatus* does not show this preference. These observations hint towards an ecological response to *C. marthae’s* skin depigmentation^76^. Therefore, this species might be found in more vegetated areas also because, here, iguanas might find a more shelters-enriched environment that could facilitate their basking-regulation behavior^77^.

Our study outlines that small-scale spatial pattern, both in terms of habitat productivity and of an undetermined spatial effect revealed by the tensor product smooth on capture locations coordinates, did affect the isotopic composition of samples (Table 1—Model 2, Model 3). On the one hand, we argued that this finding supports differential use of microhabitats by these two species. On the other hand, as the isotopic composition recorded for these species should reflect a timescale of a few to several months, this result strongly suggests that, at least in the time of the year for which our data may be informative, the feeding ranges of individuals of these two species may be extremely restricted. More in general, as our study only covered an area of *ca.* 2 km^2^, these results highlight the importance of considering the spatial dimension of processes when assessing ecological problems even at relatively small spatial scales.

## Conclusions

In this study, we evaluated, for the first time, trophic competition between the critically endangered *C. marthae* and the syntopic *C. subcristatus* on Wolf Volcano. Competition was quantitatively estimated, and a mechanism of resource partitioning was described, through which the two congeneric species may coexist. Some trophic niche partitioning is also likely to exist between sexes, especially in *C. subcristatus*. Differences in isotopic composition between species and sexes are largely (but not entirely) explained by differential use of microhabitats and body size differences between and within species. While the indications of niche partitioning mechanisms between the two species suggest potential for coexistence of these two endemic iguanas, the observed extent of niche overlap raises concern over the impact that interspecific competition may have on the only known population of *C. marthae*. This urgently calls for a careful evaluation of conservation actions aimed at sustaining the only existing population which may prudentially include the creation of a new, viable population in a sanctuary area, in another suitable area of the archipelago. Finally, this study underlines the importance of considering small-scale spatial mechanisms when working in an ecological framework.

## Supplementary Information

Supplementary Information.

## Data Availability

The data are available upon reasonable and documented request to the senior author.

## Code availability

Code is available upon reasonable request.
