# Supplementary material for: Nitrogen and carbon stable isotope analysis sheds light on trophic competition between two syntopic land iguana species from Galápagos
Source: Sci Rep. 2022 Oct 7;12:16897. doi: 10.1038/s41598-022-21134-2 (PMC9546867; doi:10.1038/s41598-022-21134-2)
Supplement: Supplementary file 1 — Supplementary Information. [file 41598_2022_21134_MOESM1_ESM.docx]

**Supplementary material**

**Nitrogen and carbon stable isotope analysis sheds light on trophic competition between two syntopic land iguana species from Galápagos**

Marco Gargano^1^, Giuliano Colosimo^2,*^, Paolo Gratton^2^, Silvio Marta^3^, Mauro Brilli^4^, Francesca Giustini^4^, Christian Sevilla^5^ & Gabriele Gentile^2^

^1^PhD Program in Evolutionary Biology and Ecology, Department of Biology, University

of Rome Tor Vergata

^2^Department of Biology, University of Rome Tor Vergata, Via della ricerca scientifica, snc - 00133 Rome, Italy

^3^Department of Environmental Science and Policy, Università degli Studi di Milano, via Celoria 10, 20133 Milano, Italy

^4^Italian National Research Council, Institute of Environmental Geology and Geoengineering IGAG - CNR, Area della Ricerca di Roma1, Via Salaria km 29.300, I-00015 Monterotondo Stazione, Rome, Italy

^5^Galápagos National Park Directorate, Av. Charles Darwin - 200102 Puerto Ayora, Is. Santa Cruz, Galápagos, Ecuador

*Corresponding author: Giuliano Colosimo, [giuliano.colosimo@uniroma2.it](mailto:giuliano.colosimo@uniroma2.it)

ORCID:

Marco Gargano: 0000-0003-0073-9094

Giuliano Colosimo: 0000-0002-0485-9758

Paolo Gratton: 0000-0001-8464-4062

Silvio Marta: 0000-0001-8850-610X

Gabriele Gentile: 0000-0002-1045-6816

Mauro Brilli: 0000-0002-2536-5714

Francesca Giustini: 0000-0002-7159-469X


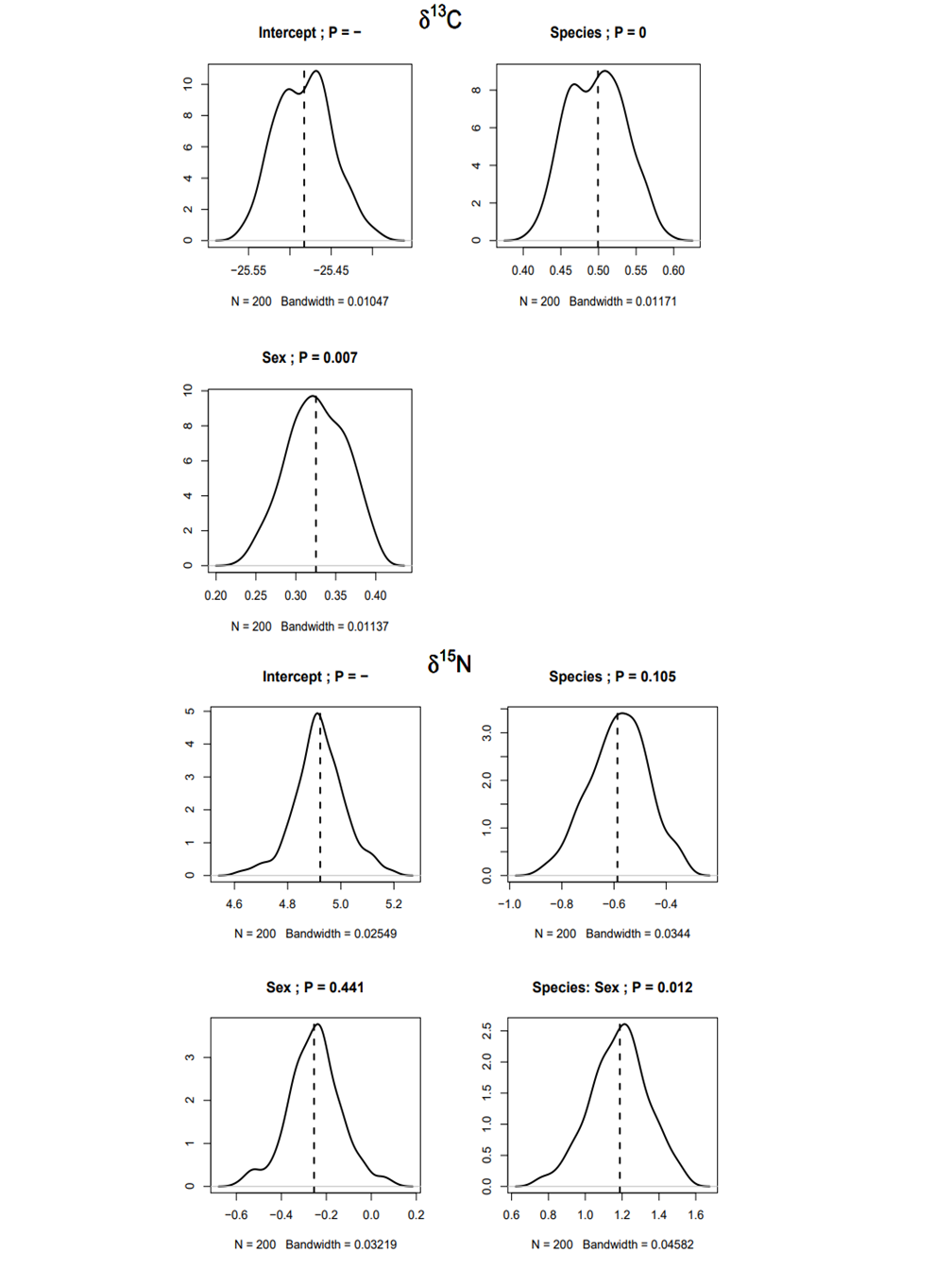


**SM Figure 1** Result of stability procedure for bivariate linear model with δ^13^C and δ^15^N as response variables and species and sex as predictors. The figure shows the distribution of the coefficients obtained after re-fitting the model 200 times on random sub-samples with 90% of the data. Dashed line indicates the coefficient obtained with the full model. The title of each graph shows the variable considered and the corresponding *P*-value in the full model.


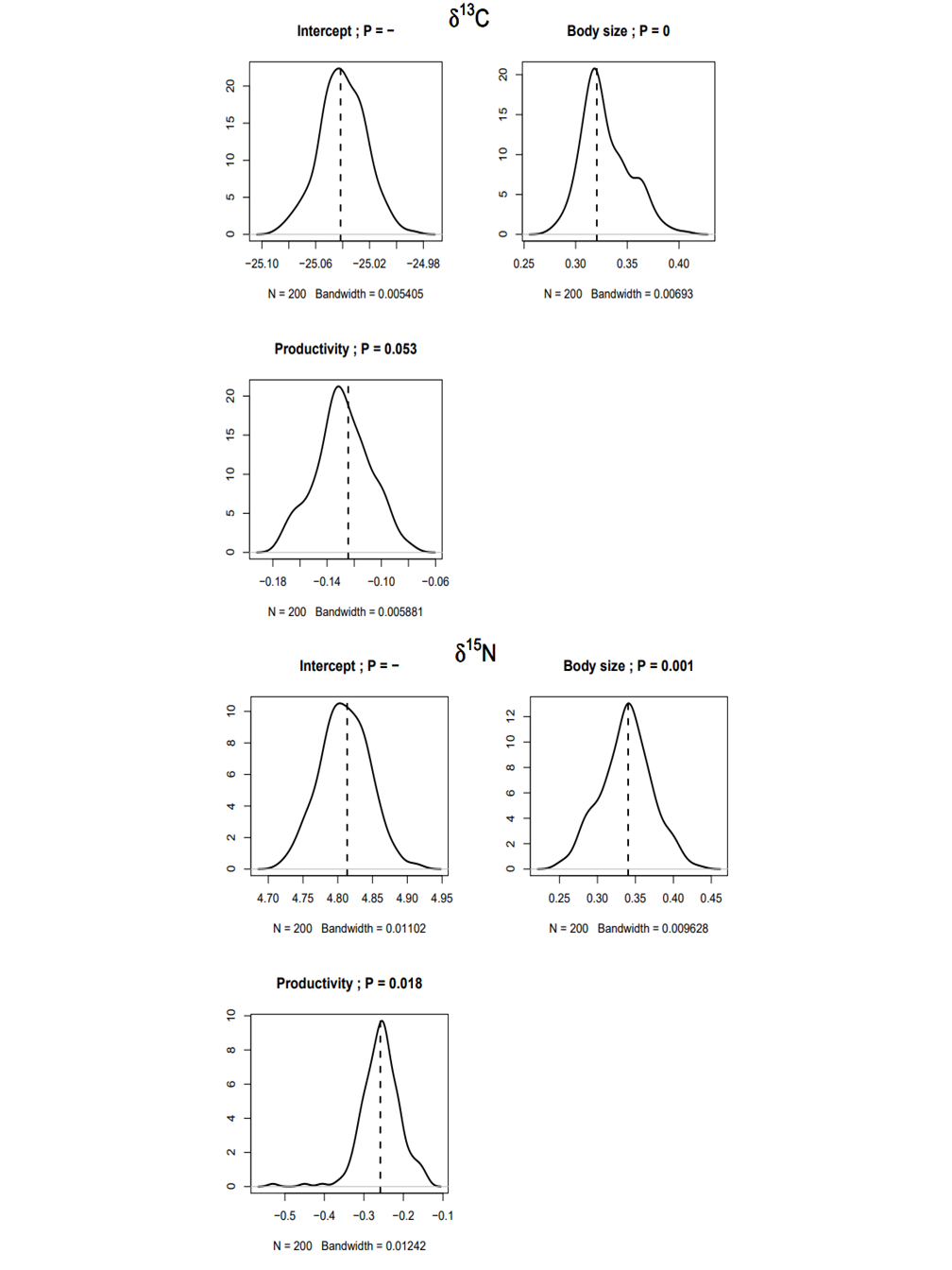


**SM Figure 2** Result of stability procedure for generalized additive model with δ^13^C and δ^15^N as response variables and body size and productivity as predictors. The figure shows the distribution of the coefficients obtained after re-fitting the model 200 times on random sub-samples with 90% of the data. Dashed line indicates the coefficient obtained with the full model. The title of each graph shows the variable considered and the corresponding *P*-value in the full model.


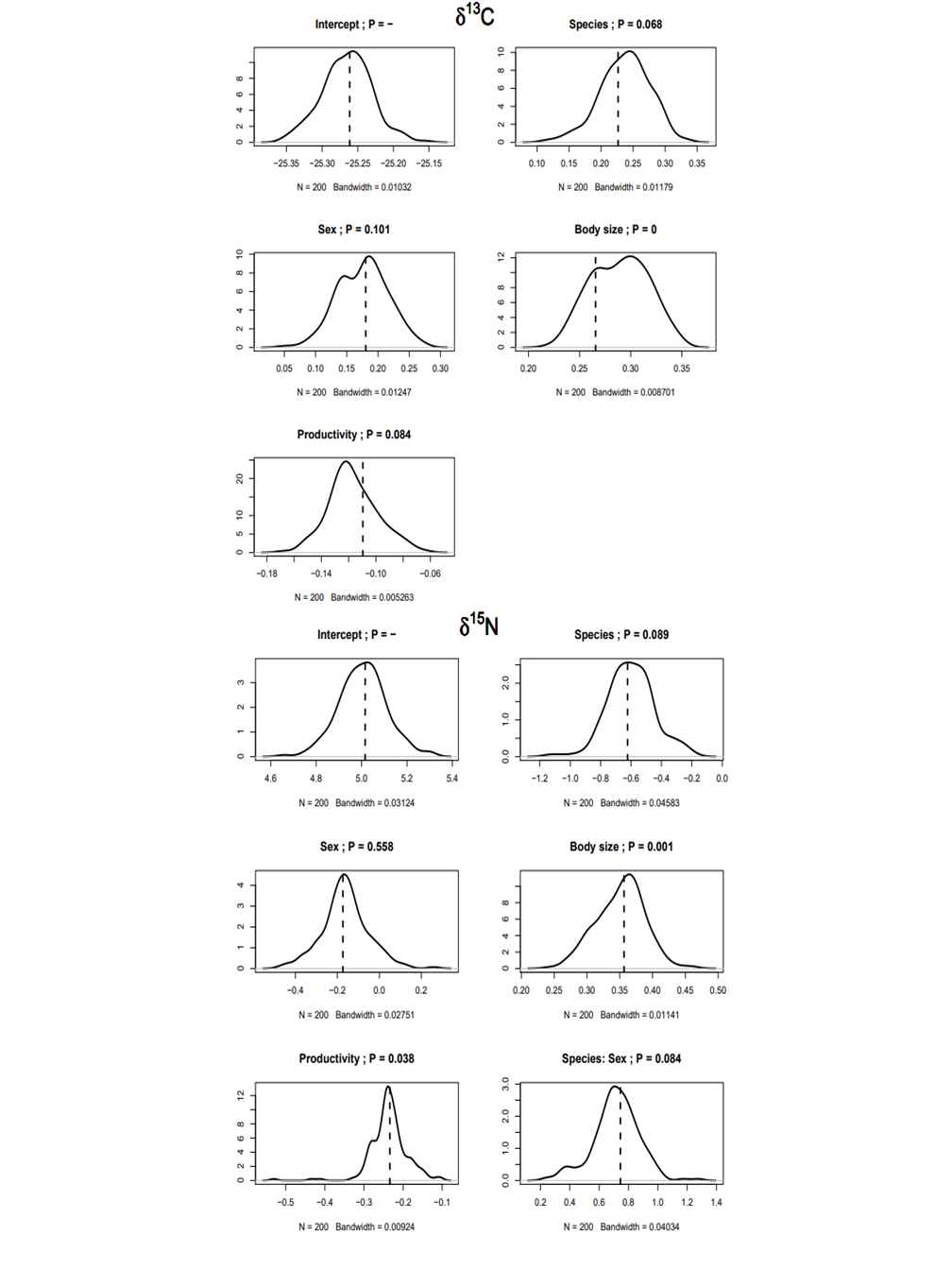


**SM Figure 3** Result of stability procedure for generalized additive model with δ^13^C and δ^15^N as response variables and species, sex, body size and productivity as predictors. The figure shows the distribution of the coefficients obtained after re-fitting the model 200 times on random sub-samples with 90% of the data. Dashed line indicates the coefficient obtained with the full model. The title of each graph shows the variable considered and the corresponding *P*-value in the full model.


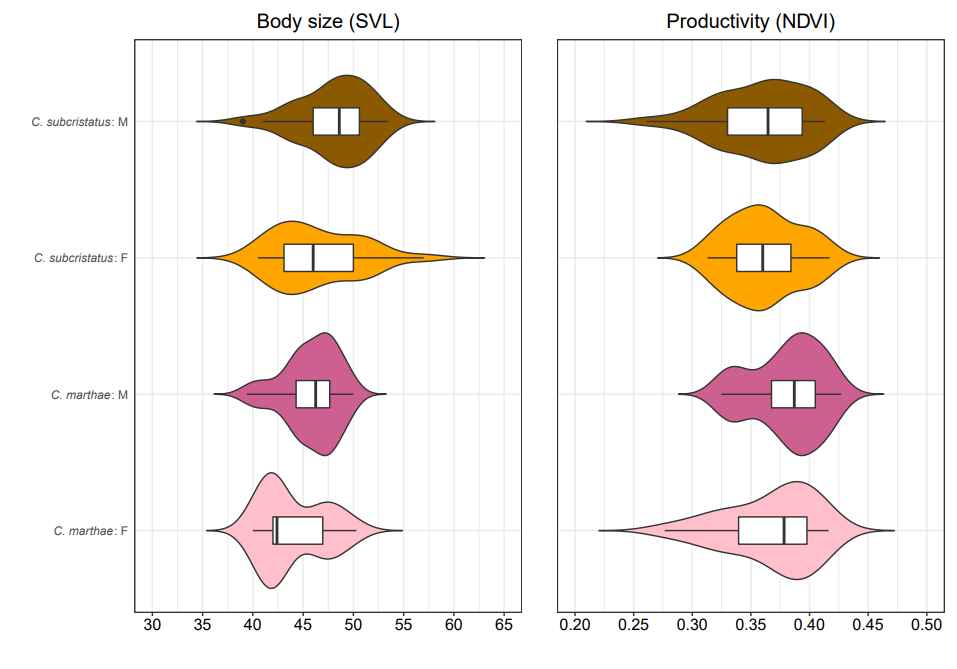


**SM Figure 4** Violin plots for body size and productivity of the capture points between and within the species. This figure shows the differences in body size (expressed as snout-to-vent-length, SVL, in cm) and productivity of the capture points (expressed as Normalized Difference Vegetation Index, NDVI) between sex classes of each species (*Conolophus subcristatus* and *Conolophus marthae*).


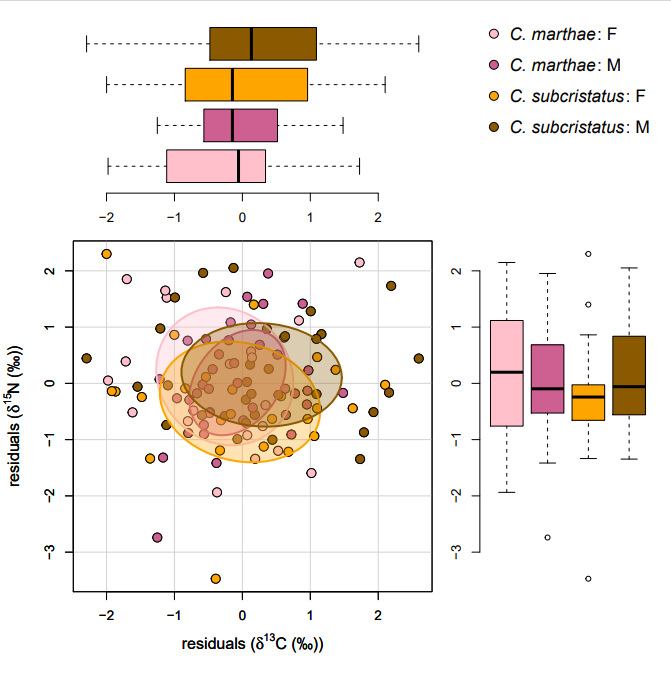


**SM Figure 5** δ^13^C, δ^15^N residuals biplot illustrating the isotopic niche of *C. subcristatus* and *C. marthae* males and females after the effects of body size, productivity and space were removed. Each dot represents a single individual. Solid lines enclose standard ellipse areas with sample size correction (SEA_c_). Marginal boxplots show the distribution of δ ^13^C and δ ^15^N residuals for each combination of sex and species. Color codes are shown in the top right corner.
